# Antioxidant, Antimicrobial, and Anticancer Activity of Basil (*Ocimum basilicum*)

**DOI:** 10.3390/antiox14121469

**Published:** 2025-12-07

**Authors:** Efthymios Poulios, Sousana K. Papadopoulou, Evmorfia Psara, Constantinos Giaginis

**Affiliations:** 1 Department of Food Science and Nutrition, School of Environment, University of Aegean, 81400 Lemnos, Greece; fnsd21013@fns.aegean.gr; 2 Department of Nutritional Sciences and Dietetics, School of Health Sciences, International Hellenic University, 57001 Thessaloniki, Greece; souzpapa@gmail.com

**Keywords:** basil, phenolic acids, flavonoids, antioxidant, DPPH, ABTS, FRAP, biofilm, bacteria, apoptosis

## Abstract

**Background/Objectives:** For many years, herbs and spices have been used, due to their aroma and flavor, in the food industry and cuisine. It is also well known that phytochemicals from these plant parts have many health benefits and are used for the prevention and treatment of many human diseases. Basil (with the most representative species *Ocimum basilicum*) is a perennial herb with a characteristic aroma, containing many bioactive components such as phenolic acids, flavonoids, tannins, saponins, alkaloids, polysaccharides, vitamins, proteins, amino acids, and essential oils, with beneficial effects on human health. The aim of this study is to review the antioxidant, antimicrobial, and anticancer activity of basil, according to recent literature. **Methods:** A thorough search in the international databases (Scopus, PubMed, Google Scholar, and Web of Sciences) was conducted from January 2015 to October 2025, using characteristic keywords in combinations. **Results:** Bioactive components of basil show a significant antioxidant activity, as detected by radical scavenging activity (measured by the 2,2-diphenyl-1-picrylhydrazyl (DPPH), 2,2′-azino-bis-(3-ethylbenzothiazoline-6-sulfonic acid (ABTS), ferric reducing antioxidant power (FRAP), oxygen radical absorbance capacity (ORAC) assays), activation of antioxidant enzymes (glutathione peroxidase (GPX), superoxide dismutase (SOD), catalase (CAT)), enhancement of reduced glutathione (GSH) and reduction in malondialdehyde (MDA) and thiobarbituric acid-reactive substance (TBARS) levels, and protection of cells from hydrogen peroxide (H_2_O_2_)-toxicity. Additionally, inhibition of growth and cell death of many Gram-positive and Gram-negative bacteria strains, maintained by cell membrane damage, inhibition of efflux pumps, as well as inhibition of biofilm formation, anti-protozoan, antifungal, and antiviral activities, have been noticed for basil bioactive components. A synergism with antibiotics has also been reported. Finally, anticancer activity has been reported, according to apoptosis induction, cell cycle arrest, anxiety reduction, and health improvement of cancer patients. **Conclusions:** Basil bioactive components have been reported for their high antioxidant, antimicrobial, and anticancer properties. However, future studies, especially at the clinical level, are strongly proposed in order to unravel the significant role of basil in human health and the safety of its bioactive components in healthcare usage.

## 1. Introduction

Herbs are typically the leaves of aromatic plants, which are separated from roots, seeds, and other organs of plants. On the other hand, spices are the remaining parts after drying of plants’ organs except leaves [1]. For many centuries, herbs and spices have been used both for culinary purposes, due to their aroma and flavor, and for medicinal purposes too. Protection from acute and chronic diseases and a high therapeutic potential of these plant parts have been reported. Many health benefits, such as antioxidant, anticancer, antimicrobial, antidiabetic, anti-inflammatory, and anti-lipidemic and cardioprotective properties, have been shown. These benefits are especially due to products of their metabolism, especially the secondary metabolites, named as phytochemicals [2]. Phytochemicals are categorized in four main groups: phenolic components (phenolic acids and flavonoids), alkaloids, terpenoids, and sulfur-containing compounds [3,4]. A series of different methods has been employed in order to extract, purify, and measure the bioactivity of these phytochemicals [5,6]. Many diseases and pathological situations, which can be prevented or co-treated with many phytochemicals have been extensively reported, including oxidative stress [1,7,8], inflammation [9,10], neurodegenerative disorders [11,12], diabetes [13,14], hypertension [15,16], cardiovascular diseases [17,18], microbial infections [19,20], COVID-19 [21,22], obesity [23,24] and cancer [25,26].

Reactive oxygen species (ROS), parts of which are superoxide anion (O_2_.^−^), hydrogen peroxide (H_2_O_2_), and hydroxyl radical (HO^•^), are formed by the partial reduction of oxygen in aerobic metabolism. When ROS production is increased, or their removal is decreased, a pathological situation called oxidative stress is formed, leading to DNA, protein, and lipid damage, resulting in many diseases such as neurodegenerative disorders, cancer, cardiovascular diseases, and aging [27]. In order to defend against oxidative stress, a series of endogenous enzymes, such as SOD, CAT [28], GPX [29], thioredoxin (Trx), and glutaredoxin (Grx) [30,31], acts in order to reduce ROS or inhibit their production. However, this defense is not always enough. On the other hand, plant secondary metabolites react as general antioxidants. Chemical classes such as phenolic acids, flavonoids, stilbenes, lignans, tannins, and vitamins A, E, and C, due to their chemical type, can reduce or terminate oxidant molecules and act complementary to endogenous antioxidant enzymes [32].

Microbial infections are a huge sanitary problem that can cause many diseases. Antibiotics, chemical substances produced by microorganisms, causing growth inhibition and cell death of other microorganisms, have been used for many years. However, the development of many multidrug-resistant (MDR) microorganisms, such as bacteria, fungi, and protozoa, makes antibiotic usage less effective. Recent years’ research focuses on the antibacterial, antiviral, antifungal, and antiprotozoan activity of plant secondary metabolites, which can be used either as antimicrobial agents or in combination with known antibiotics, antifungal, and antiviral agents [33].

Cancer is the second death-leading disease, after cardiovascular diseases, worldwide. It is characterized by the uncontrolled growth of cells, caused by genetic (gene mutations), epigenetic, and environmental factors. The most common cancers are lung, breast, stomach, colon, skin, and prostate cancer. Treatment of cancer includes chemotherapy (including cytotoxic drugs), surgery, radiotherapy, and immunotherapy. Due to unwanted consequences to normal cells, MDR of many cancer cells, and recurrence of cancer, research is focused on natural products having anticancer activity, such as growth-inhibition or apoptosis-induction, without affecting normal cells [26,34].

Basil, also called great basil or sweet basil (*Ocimum basilicum* L.), is a common culinary herb, belonging to the Lamiaceae family, native to tropical regions from Central Africa to Southeast Asia, and cultivated in temperate and Mediterranean regions, such as Europe. Other similar species include Camphor basil or African basil (*O. kilimandscharicum*), clove basil, also known as African basil (*Ocimum gratissimum*), and holy basil (*Ocimum tenuiflorum*, formerly known as *O. sanctum*). Morphologically, it is an annual or sometimes perennial herb, 30–150 cm in height, glossy, with ovulate green or purple leaves and small and white flowers. It has been used for many years for its aroma and flavor added to foods. Basil also has many health benefits, as reported by its preservative and therapeutic effects [35], and has been used for centuries for the treatment of fever, flu, colds, and the improvement of reproduction, digestion, and respiration [36]. Mechanisms of health improvement include antioxidant, anti-inflammatory, anti-aging, wound healing, antidiabetic, cardioprotective, immunity enhancement, antiulcer, anti-depressant, anticoagulant, anti-atherosclerotic, hypolipidemic, neuroprotective, anticancer, antibacterial, antifungal, and antiviral properties. These health benefits are due to a wide range of different classes of bioactive molecules, especially the volatile compounds contained in the basil essential oil of leaves and seeds. These classes include phenolics (rosmarinic acid, and cichoric acid), flavonoids (linalool, eucalyptol, estragole, trans-α-bergamotene, 1,8-cineole, neryl acetate, geraniol, and methyl eugenol), tannins, saponins, alkaloids, steroids, proteins, lipids (linoleic and linolenic fatty acids), reducing sugars, glycosides, polysaccharides, dietary fiber, and minerals (magnesium, potassium, and calcium) [35,36,37,38,39].

In this study, we present a comprehensive review of the antioxidant, antimicrobial, and anticancer properties of basil (especially the species *Ocimum basilicum*), according to the available scientific literature.

## 2. Methods

A thorough search was conducted in the international databases (Scopus, PubMed, Google Scholar, and Web of Sciences), from January 2015 to October 2025, using characteristic keywords, alone and/or in combinations, such as basil OR Ocimum basilicum AND antioxidant (99 results) OR antimicrobial (41 results) OR anticancer (9 results). More of the previous articles were also used for the introduction. We only included studies written in the English language, research articles, clinical trials, and systematic reviews, focusing on antioxidant, antimicrobial, and anticancer effects of basil. After exclusion of some publications, we finally used 50 articles. All authors acted as reviewers in order to enhance the validity.

## 3. Results

### 3.1. Antioxidant Activity of Basil

Basil is extensively studied for its antioxidant activity due to its bioactive components (Table 1). Most studies present results from in vitro experiments. Different parts of the plant, different extraction methods, and different solvents used for extraction show different results. In a recent study, hydroalcoholic extract of basil leaves showed antioxidant activities, as measured by the DPPH, reducing power, H_2_O_2_ scavenging, and anti-lipid peroxidation assays, which varied in the inhibitory concentration 50 (IC50) values, according to the specific in vitro method used [40]. In another study, phytochemical characterization of a polyphenolic fraction of basil, by using ultra-performance liquid chromatography (UPLC) with a diode-array detector (DAD) and mass spectrometry (MS), revealed a high concentration of caffeic acid derivatives, especially chicoric and rosmarinic acid. This fraction showed significant antioxidant activity in human normal colon epithelial cells and colorectal adenocarcinoma cells, as measured by the DPPH and FRAP assays, the activation of SOD and CAT enzymes, and the reduction in MDA levels after oxidative stress induction. Additionally, a cytoprotective effect in human normal colon cells after H_2_O_2_ addition was also detected [41]. More to the point, Nadeem and colleagues indicated that ethanolic extracts from basil showed the highest concentration of flavonoids, phenolic acids, and tannins, especially ellagic acid, rosmarinic acid, liquiritigenin, catechin, and umbelliferone, as analyzed by liquid chromatography–electrospray ionization-tandem mass spectrometry (LC-ESI-MS/MS), and the highest antioxidant activity as measured by the DPPH, FRAP and H_2_O_2_ assays, as compared to n-hexane, dichloromethane and water extracts [42].

In a similar study, extracts of basil aerial parts by using dichloromethane, ethanol, and sunflower oil, with re-extraction using acetonitrile, were prepared. Fatty acids were detected in all extracts, whereas phenolic acids and flavonoids were dominant in ethanolic extracts, diterpenoids in acetonitrile extracts, and triterpenoids in dichloromethane extracts, as detected by high-performance liquid chromatography diode-array detector/electrospray ionization time-of-flight (HPLC-DAD/ESI-ToF-MS). Ethanolic and dichloromethane extracts showed higher antioxidant activity, as measured by the scavenging of DPPH radical. These differences were due to the different polarities of solvents used [43]. Similarly, Abdel-Razakh and colleagues showed a high total phenolic content (TPC) in ethanolic extracts and solvent fractions of *Ocimum basilicum*, whereas the highest TPC, total flavonoid content (TFC), and total tannin content (TTT) were detected in the ethyl acetate fractions. Rosmarinic acid was the dominant component found. Additionally, DPPH and ABTS assays showed that the ethyl acetate fraction has the strongest antioxidant activity [44]. More to the point, Mahmood and colleagues investigated the antioxidant activity of sweet basil residues, after drying in a microwave, and extraction by using hydrodistillation, headspace solid-phase microextraction (HS-SPME), and analysis by gas chromatography–mass spectrometry (GC-MS). Methylcinnamate, β-linalool, methyleugenol, and estragole were the dominant components found in raw material, rosmarinic acid and luteolin in the microwave-dried sample, whereas a decrease in methyl-eugenol levels was found in oven- and microwave-dried samples. Microwaved samples showed the highest TPC and antioxidant activity, as measured by the Folin–Ciocalteu and DPPH assays, respectively [45]. In a similar study, Quamar and colleagues used GC-MS of non-polar basil leaf extracts and found significant levels of monoterpenes, sesquiterpenes, triterpenes, hydrocarbons, phthalates, phyrosterols, and fatty acids. Bioactive components mainly detected were terpineol, linalool, methyl linolenate, tau-cadinol, methyl palmitate, palmitic acid, and linolenic acid. Additionally, by using electrospray ionization–high-resolution tandem mass spectrometry/mass spectrometry (ESI-HR-MS/MS), they detected phenolic acids, alkaloids, flavonoids, amino acids, lignin, coumarin, and terpenes in the polar extracts. Acetonic extracts showed the highest flavonoid and phenolic content among others, whereas all extracts showed high antioxidant activity as measured by ABTS and DPPH assays, with the dichloromethane extract showing the highest activity. More to the point, ethyl acetate, dichloromethane, and acetone extracts inhibited 2,2′-azobis(2-amidinopropane) dihydrochloride (AAPH)-induced oxidation of human erythrocytes [46].

More to the point, Siripongvutikorn and colleagues, by using liquid chromatography–electrospray ionization-quadrupole time-of-flight-mass spectrometry (LC-ESI-QTOF-MS/MS), identified different bioactive components in spicy basil leaf curry, mainly represented by hydroxycinnamic acid, quininic acid, betulinic acid, luteolin, catechin, gingerol, eugenol, and kaempferol. Antioxidant activity, as measured by DPPH, ABTS, FRAP, and ORAC assays, was mainly gained by the presence of luteolin-7-O-glucoside and cymaroside [47]. Additionally, Złotek and colleagues reported acetonic extraction of phenolic components of fresh and freeze-dried basil leaves, with a high concentration of acetic acid, as the best extraction condition, as compared to methanolic extraction. When the procedure was repeated three times, as compared to one shaking, it offered higher concentrations of phenolic components obtained from fresh basil leaves, whereas this was not the case when frozen leaves were used. No difference in the two procedures was obtained when lyophilized leaves were used. High concentration of phenolic components was correlated with high antioxidant activity [48].

Moreover, Gupta and colleagues reviewed the role of basil in skin health, with emphasis on its antioxidant activity. Many bioactive components, such as phenolic acids, flavonoids, and terpenes, were reported to neutralize ROS and reactive nitrogen species (RNS), and inhibit the production of free radicals, acting as an efficient antioxidant herb. More to the point, these bioactive components inhibit central pathways of inflammation, such as mitogen-activated protein kinase (MAPK), cyclooxygenase-lipoxygenase (COX-LOX), and nuclear factor kappa-b (NF-kB) pathways, by reducing proinflammatory cytokine production and activity. These antioxidant and anti-inflammatory properties protect skin from aging. According to these findings, skin pathological conditions like eczema and acne could be efficiently treated by basil bioactive components [49].

Essential oils of basil have also been mentioned for their antioxidant activity, due to the variety of bioactive compounds they include. Yibeltal and colleagues identified a high antioxidant activity of basil leaf essential oil, as measured by the DPPH, ascorbic acid, and hydrogen peroxide radical scavenging assays [50]. Additionally, Eid and colleagues also showed a high antioxidant activity of *O.basilicum* seed essential oil compared with Trolox control [51]. Moreover, Park and colleagues reported the high DPPH and ABTS radical scavenging activity of basil essential oil [52]. In another study, sweet basil crude oil was processed via molecular distillation. After GC-MS analysis, linoleic acid, estragole, and methyl eugenol were mainly identified in the residue fraction, whereas the distillate fraction mainly contained α-bergamotene, α-cadinol, and methyl eugenol. Residue fraction showed a high radical scavenging activity of DPPH and ABTS, as compared to the distillate fraction [53].

Comparative studies of different basil varieties have also been reviewed. Sharma and colleagues made a comparative extraction of bioactive components of leaves of three *Ocimum* species (*Ocimum basilicum*, *O. gratissimum*, and *O. tenuiflorum*) by using water, acetone, methanol, and ethanol. Acetonic extracts of all species contained the highest concentration of flavonoids, tannins, and phenolic acids, whereas *O. basilicum* showed the highest antioxidant activity [54]. Additionally, Araújo Couto and colleagues reported the inhibition of the linoleic acid peroxidation and DPPH radical scavenging by essential oils from 24 different basil genotypes, attributed mainly to eugenol and the synergism between minor compounds [55]. More to the point, Fayezizadeh and colleagues analyzed the chemical composition (polyphenols, anthocyanins, flavonoids, and vitamin C) and antioxidant activity of different genotypes and cultivars of basil microgreens. Persian Ablagh genotypes Violeto and Kapoor showed the highest antioxidant potential composite index (APCI). Chemical composition and antioxidant activity varied according to the temperature conditions of culture [56]. More to the point, Mahendran and Vimolmangkang extracted essential oils of *Ocimum basilicum* and *Ocimum americanum* leaves by steam distillation. GC-MS analysis detected mainly limonene, longifolene, camphor, isoledene, and caryophyllene in *O. americanum* leaf essential oil, whereas camphor, citral, estragole, and caryophyllene were mainly found in *O.basilicum* leaf essential oil. Additionally, the antioxidant activity of *O. basilicum*, as analyzed by the DPPH, FRAP, and ABTS assays, was more potent in comparison to *O. americanum* [57]. In a similar study, ethanolic extracts of germinated *O. basilicum* and *O. gratissimum* seeds exhibited higher concentrations of phenols and flavonoids than other extracts did, resulting in superior antioxidant potential with substantially lower half-maximal inhibitory concentration (IC50) values for scavenging ABTS and superoxide anion radical, and for inhibiting lipid oxidation, in comparison to other extracts [58]. In another comparative study by Tenore and colleagues, an evaluation of the antioxidant activity of 2 varieties of “Napoletano” green and purple basil (*Ocimum basilicum*) was performed. All varieties contained a high polyphenolic content, whereas purple basil was characterized by higher ferric-reducing and radical-scavenging activity, probably connected to its high anthocyanin content [59]. In another study, steam distillation of sweet basil (*Ocimum basilicum*) and holy basil (*Ocimum tenuiflorum*) resulted in higher content and different concentrations of bioactive components of essential oil as compared to hydrodistillation. On the other hand, the methyl eugenol concentration of *O. tenuiflorum* essential oil, as well as methyl cinnamate and estragole concentrations of *O. basilicum* essential oil, were higher after hydrodistillation. No difference was observed in the antioxidant activity between the two types of extraction used [60].

Nanosized emulsions of basil extracts have also been investigated. In a recent study from Manzoor and colleagues, nanosized basil essential oil in microemulsions, in order to improve their water solubility, stability, volatility, and bioavailability, showed high antioxidant activity in a dose-dependent manner [61].

Moreover, Abidoye and colleagues investigated the antioxidant activity of a drink composed of roselle calyces (*Hibiscus sabdariffa*) and sweet basil leaves (*Ocimum basilicum*). Addition of basil reduced the lycopene and vitamin C concentration, whereas it increased the total carotenoid concentration and the radical scavenging activity, as measured by the oxygen radical absorbance capacity (ORAC) and ABTS assays. Higher temperature storage (29 °C), as compared to 4 °C, improved the antioxidant activity [62].

There are a few research articles in the literature focused on the antioxidant activity of basil. Othman and colleagues investigated the antioxidant properties of Hail *Ocimum* extract and its flavonoids against hepatorenal damage in experimental rats after a high-fat diet (HDF) and streptozotocin treatment. Basil extract or basil flavonoids co-treatment resulted in GSH levels increase and SOD, CAT, GPX, and glutathione reductase (GR) enzymes activity induction [63]. Additionally, Eftekhar and colleagues found that basil extract decreased the tracheal bronchoalveolar lavage fluid levels of oxidant markers of rats sensitized to methacholine and ovalbumin, whereas levels of antioxidant markers were increased, in a dose-dependent manner [64]. Finally, Ben Mansour and colleagues found that a methanolic extract of *O. basilicum* seeds offered protection in the kidneys of adult rats, after carbon tetrachloride (CCI4)-induced oxidative stress. More specifically, basil extract reduced the oxidative-stress kidney serum markers, like urea, creatinine, TBARS, protein carbonyls, and lipid peroxidation, whereas antioxidant enzyme activity, like GPΧ, SOD, catalase, and GSH levels, was elevated [65].

In conclusion, basil extracts contain many bioactive components with high antioxidant activity. Different parts of basil, different solvents, and extraction methods, as well as different antioxidant assays, obtain various concentrations of bioactive components and levels of antioxidant potential.

### 3.2. Antimicrobial Activity of Basil

The antimicrobial (antibacterial, antiparasitic, and antifungal) activity of basil (Table 1) has been extensively reported in the literature [66]. Most of the studies have been made in vitro and include basil extracts by using different solvents and different extraction and purification methods. Vijay and colleagues indicated a dose-dependent antifungal activity of a hydroalcoholic extract of basil leaves, by using a time-killing curve assay and a modified poison food assay [40]. Moreover, ethanolic, dichloromethane, and sunflower oil re-extracted with acetonitrile extracts of basil aerial parts showed high antifungal activity, whereas antibacterial activity was detected in ethanolic and acetonitrile extracts, as shown by the broth microdilution method [43]. Additionally, ethanolic and aqueous seed extracts of *Ocimum basilicum* inhibited the growth of anaerobic periodontal pathogens in different concentrations. The inhibition zone was wider by using the aqueous extract. Both extracts showed a statistically significant antibacterial effect, as compared to chlorhexidine gluconate [67]. Moreover, Alsalamah and colleagues identified steroids, glycosides, tannins, and flavonoids in extracts of *Ocimum basilicum* leaves, as well as steroids, flavonoids, and saponins in seed extracts. Anibacterial activity, as observed by the disk diffusion and direct contact assays, against *Escherichia coli*, *Staphylococcus aureus*, and *Pseudomonas aeruginosa*, was measured. Leaf extracts were reported to have higher antibacterial activity, in comparison to seed and stem extracts [68]. More to the point, Backiam and colleagues reported a strong antimicrobial activity of ethanolic and methanolic extract of *Ocimum basilicum*, containing carbohydrates, proteins, saponins, quinones, tannins, alkaloids, flavonoids, and phenolic acids, without steroids and terpenoids, against vancomycin-resistant (VRE) enterococcal strains and microbial type culture collection (MTCC) strains. Total flavonoid and phenolic content were higher in the ethanolic extract, followed by the methanolic and aqueous extracts. *Staphylococcus aureus* was more susceptible to the ethanolic extract, as compared to the other MTCC strains tested. Aqueous extract showed less antimicrobial activity, whereas steroids, terpenoids, and tannin were absent. Cell membrane damage and loss of cell membrane integrity were reported as the main mechanisms of the antimicrobial activity [69].

Basil essential oils, which contain a variety of bioactive components, have also been reported for their antimicrobial activity. In a recent study, *O. basilicum* seed essential oil showed a high antibacterial activity against vaginal pathogens, such as *Escherichia coli*, *Staphylococcus aureus*, *Klebsiella pneumoniae*, *Pseudomonas aeruginosa*, *Proteus mirabilis*, and antifungal activity against *Candida albicans* [51]. In a similar study, basil essential oil contained mainly monoterpene, sesquiterpene, and phenylpropene derivatives, as analyzed by GC-MS, and inhibited the growth of *Chryseobacterium gleum*, *Gardnerella vaginalis*, and *Candida albicans*, whereas there was no toxicity observed in *Lactobacillus crispatus* or human dermal fibroblasts. Fraction 3 after HPLC preparation showed a high concentration of methyl trans-cinnamate, correlated with the highest antimicrobial activity [52]. More to the point, Yibeltal and colleagues showed that essential oil of basil flower represents the higher antibacterial activity against *Staphylococcus aureus* ATCC-25923, as measured by zone of inhibition, minimum inhibitory concentration (MIC) and minimum bactericidal concentration (MBC), in comparison to other bacteria strains tested, whereas the maximum zone inhibition, MIC and minimum fungicidal concentration (MFC) was reported for the basil leaf essential oil against *Candida albicans* [50].

Additionally, Gupta and colleagues reviewed the antimicrobial activity of basil essential oils, including estragole, eugenol, and linalool, focusing on the microbial cell membranes disruption, the increase in membrane permeability, the intracellular leakage of bacterial cells, and the inhibition of biofilm formation. Due to these beneficial effects, basil is used in dermatological and cosmetic formulations, like skincare, haircare, and dentalcare [49]. In addition, microemulsion formulations of basil essential oils showed higher antimicrobial activity against *Staphylococcus aureus* and *E. coli*, at lower concentrations in comparison with classical essential oils of basil [61]. Moreover, Yaldiz and colleagues showed that ethanol extract and essential oil of *Ocimum basilicum* have antibacterial and anti-quorum-sensing activity against many Gram-positive and Gram-negative bacterial species, whereas antifungal effects against *C. albicans* were also observed. Microorganism genotypes of PI 253157, PI 296390, PI 296391, PI 414199, PI 531396, PI 652071, midnight, and Dino cultivars were more susceptible to the essential oil. The highest anti-quorum-sensing effect was reported in PI 172997, PI 190100, PI 296391, PI 414199, and PI 652070 genotypes, as well as Moonlight and Dino cultivars. A relationship between different genotypes, depending on microorganisms and anti-quorum-sensing activity, was shown by a dendrogram. PI 207498, P379412, and Ames 29184 genotypes were categorized in the same group. Finally, 47% of the total variation was found in all forms [70].

In silico studies have also been reported. In a study from Putri and colleagues, nevadensin, a flavonoid found in basil, has been reported for its moderate antibacterial activity against *Streptococcus mutans*, which causes dental abnormalities by forming biofilms, as measured by the MIC and MBC values, and the binding activity against the bacterial proteins Ag I/II, SrtA, and gbpC, whereas in silico studies showed that it has the same binding strength as chlorhexidine in SrtA inhibition, and a weaker binding affinity to Ag I/II and gbpC [71]. In another study of the antibacterial activity of *O. americanum* leaves extract by n-hexane against *Streptococcus mutans* and *Streptococcus sanguinis*, lauric acid was reported to be the main component with antibacterial activity. MIC and MBC showed a high antibacterial activity for both strains tested. Moreover, lauric acid inhibited the MurA bacterial enzyme, which catalyzes peptidoglycan formation in the cell wall, with a higher binding activity than fosfomycin, as shown by an in silico study [72].

Combination studies with basil extracts combined with antibiotics, in order to investigate synergistic or antagonistic effects, have also been reported. Araújo Silva and colleagues investigated the antibacterial effects of *Ocimum basilicum*, in combination with antibiotics, against *Pseudomonas aeruginosa* and *Staphylococcus aureus*. The essential oil of basil showed respective MIC against *S. aureus*. A combination of the essential oil with the antibiotic imipenem showed a synergistic effect, whereas a combination with ciprofloxacin showed antagonistic effects. A combination with imipenem showed an additive effect for ATCC strains of *P. aeruginosa* and synergistic effects for the clinical strain. Additionally, a combination with ciprofloxacin has synergistic effects for clinical strains of *P. aeruginosa* [73]. More to the point, El-Samahy and colleagues constructed lignin nanoparticles with the ethanolic extract of *Ocimum basilicum*, which inhibited the growth of the bacterial strain *Salmonella* Typhimurium and the fungal strain *Trichophyton rubrum*, as measured by the MIC values, after infection of a rat and a guinea pig model, respectively. The nanoparticles also downregulated the efflux pump genes of acrB and ramA. Oral administration of these nanoparticles in rats, in combination with the antibiotic ciprofloxacin, showed a positive effect in blood, kidney, and liver parameters, a decrease in oxidative stress markers and MDA levels, and an increase in total antioxidant activity, whereas an elevation of *Salmonella* clearance in the liver and intestine was also observed. Additionally, when *T. rubrum*-infected guinea pigs were treated with these nanoparticles, combined with itraconazole topically, a reduction in culture and microscopy results, and in lesion scores was observed [74].

Comparative studies between different varieties and species of basil, focusing on their antimicrobial activity, have also been reviewed. Tenore and colleagues reported a high antimicrobial activity of Napoletano green and purple basil against many human pathogenic and foodborne microorganisms [59]. Additionally, ethanolic extracts from *Ocimum gratissimum* and *Ocimum basilicum* seeds showed higher antimicrobial activity than aqueous extracts, as measured by the inhibition zones of *Bacillus subtilis*, *Salmonella enterica*, *Vibrio parahaemolyticus*, *Escherichia coli* bacterial cultures, and inhibition of *Aspergillus flavus* growth [58]. Additionally, in a comparative study of *Ocimum americanum* and *Ocimum basilicum* leaves’ essential oils, a significant antibacterial activity was found, as measured by the disk diffusion and tube microdilution methods [57]. Furthermore, extract from *Ocimum basilicum* of the Peshawar region showed a significant lethal concentration 50 (LC50) value against the *Leishmania* parasite, and significant inhibition of two Gram-positive strains, *Bacillus subtilis* and *Clostridium perfringens* type C, in comparison to the antibiotic gentamycin. A significant inhibitory activity was also seen in Gram-negative bacteria. The highest inhibition zone was reported in the *Pseudomonas aeruginosa* strain. Finally, a high LC50 value was observed by using the brine lethality assay [75].

Finally, a study on the antiviral activity of basil extract has also been reported. Kurnia and colleagues investigated the mechanism of inhibition of the main protease of SARS-CoV2 by *Ocimum basilicum* by using the Lipinski Rule of Five. Mechanisms of molecular docking inhibition were predicted by using Autodock 4.0 tools, as well as the protox and pkcsm online web servers, in order to analyze drug likeness and absorption, distribution, metabolism, excretion, and toxicity (ADMET). Binding affinities and constant values of inhibition were measured for the basil bioactive components dihydrokaempferol-3-glucoside, apigenin-7-glucuronide, and aesculetin. Dihydrokaempferol-3-glucoside and apigenin-7-glucuronide were found to bind to the active sites of His41 and Cys145, respectively, whereas aesculetin was reported to bind to the active sites of Cys145. All these components met the predicted pharmacokinetic parameters, whereas special consideration must be given to some parameters must be considered, especially for aesculetin. One violation was found for dihydrokaempferol-3-glucoside and apigenin-7-glucuronide, whereas no violation was detected for aesculetin. All these basil components can be considered as antiviral drugs against viral protease, with dihydrokaempferol-3-glucoside and apigenin-7-glucuronide reported for higher effectiveness [76].

In conclusion, a strong antimicrobial activity of basil extracts has been extensively reported for many Gram-positive and Gram-negative bacteria, fungi, protozoan strains, and SARS-CoV-2. Different solvents used for extraction result in different concentrations of antimicrobial components and different levels of antimicrobial activity.

### 3.3. Anticancer Activity of Basil

Cancer is reported as the abnormal and non-regulated proliferation of cells, starting with a gene mutation, triggered by several factors, and can be inherited or acquired. Many herb extracts are reported to have anticancer activity, and basil has been extensively reported for its anticancer properties (Table 1). As reviewed by Perna and colleagues, antioxidant components of basil leaves show anticancer activity, as identified in aqueous extracts, as shown by growth inhibition of cancer cells, cell death induction, and cancer cell viability reduction, when used in small doses. In higher doses, they show strong antiproliferative potential, cytotoxicity, apoptosis induction, cell cycle arrest, and inhibition of tumor growth, as indicated in both in vivo and in vitro studies. Consumption of 1 to 2.5 mg/daily reduces cytokine action and improves vital activity [77].

Many in vitro studies, using cancer cell lines, have been reported. Basil extracts, obtained by using different solvents and extraction methods, have been used. Avcibasi and colleagues reported that estragole isolated from basil leaves after ethanolic extraction with HPLC, and radio-labeling with ^131^I, is successfully uptaken by human brain cancer medulloblastoma (DAOY) and glioblastoma–astrocytoma (U-87 MG) cell lines, as shown by quality control studies using thin-layer radio chromatography (TLRC), in vitro bio-affinity studies, and incorporation studies investigating the cytotoxicity of this component [78]. In another study, an aqueous extract of fresh dark purple blossoms of *Ocimum basilicum* at low temperature (0 °C), using a watery solvent, was prepared, and several concentrations were used to treat the MCF-7 breast cancer cell line. Several bioactive components were detected, such as anthraquinones, tannins, anthocyanins, amino acids, proteins, glycosides, reducing sugars, flavonoids, and volatile oils, whereas alkaloids, terpenoids, and steroids were not detected. A dose-dependent alleviation of the restraint of glucose uptake, inducing mitochondrial fission and apoptosis, was observed [79]. More to the point, secreted extracellular vesicles purified from apoplast washing fluid of *Ocimum basilicum* leaves, with a size of 100–250 nm, were used as a drug delivery system in human pancreatic cancer. When these extracellular vehicles were uptaken from the pancreatic cancer cell line MIA PaCa-2, they induced apoptosis. Apoptotic protein Bax was upregulated at the transcription and translation level [80] (Chintapula et al., 2024).

Essential oils of basil also contain anticancer components. Eid and colleagues showed a high cytotoxic activity of *O. basilicum* seed essential oil against MCF-7 and Hep3B cells, as compared to the chemotherapeutic drug doxorubicin [51].

A comparative study of *Ocimum basilicum* and *O. gratissimum* extracts presented cytostatic effects on the human breast cancer cell line MCF-7, whereas only *O. basilicum* extract was reported for cytotoxicity, even after treatment interruption, cell proliferation and metabolism inhibition, as shown by measuring lactate production, and intracellular ATP content. *O. basilicum* extract induces apoptosis, whereas *O. gratissimum* induces necrosis, after 24 h of treatment. Adenosine monophosphate (AMP)-activated protein kinase (AMPK) activation was detected by both extracts, with a more pronounced effect by *O. basilicum*, whereas mammalian target of rapamycin (mTOR) signaling pathway was activated only by the *O. basilicum* extract [81].

In silico and molecular docking studies have also been reported. Sharma and colleagues performed an in silico structure-activity relationship study on orientin, a flavonoid component found in *Ocimum sanctum* L. (*O. tenuiflorum*), known as holy basil in India, and constructed a quantitative structure-activity relationship (QSAR) model and pharmacophore mapping, in order to find out potential structurally similar analogs from database of Discovery Studio (DSv3.5, Accelrys, San Diego, CA, USA) as potential anticancer agents. Analog fenofibryl glucuronide was selected for 3-(4,5-di methyl thiazol-2-yl)-2,5-diphenyltetrazolium bromide (MTT) assay in order to study its cytotoxic activity in vitro, against cancer cells. Molecular docking studies of the enzyme quinone oxidoreductase were performed by measuring the binding affinity of orientin and its selected analog. Only 41% cell death of the liver cancer cell line HepG2 was reported at 100 μg/mL concentration (at 96 h), which characterizes orientin and its analog fenofibryl glucuronide as non- or less cytotoxic/anti-carcinogenic in these concentrations and for long-term treatment [82]. In a more recent study, Βhura and colleagues performed the molecular docking assay for basil polysaccharides, in order to find out the binding potential against different epigenetic targets of breast cancer, including histone deacetylases (HDAC), HDAC1-2, 4–8, and histone acetyltransferases (HAT). Absorption, distribution, metabolism, and excretion (ADME) studies were also conducted in order to check the drug-like properties of the basil polysaccharides. Glucosamine ring, glucosamine linear, glucuronic acid linear, rhamnose linear, glucuronic acid ring, galactose ring, mannose, glucose, and xylose showed a strong binding potential against HDAC1, HDAC2, HDAC4, HDAC5, HDAC6, HDAC7, HDAC8, and HAT, indicating these polysaccharides as potential breast cancer inhibitors [83].

Combinational studies of the anticancer activities of basil extracts, combined with conditional anticancer drugs, have also been reported in order to investigate possible synergistic effects. Feng and colleagues reported a synergistic effect of basil polysaccharides with the chemotherapeutic drug gefitinib in lung tumor growth, as shown in an immunodeficient gefitinib-resistant xenograft mouse model. Mechanisms of this action include the gut microbiota and the relative metabolites modulation, by multiple metabolic pathways, as shown by multi-omics assays, including 16S rDNA amplicon sequencing, and LC-MS. These changes probably affect cancer signaling pathways and lung-resistance related protein, which are significant in the effectiveness of epidermal growth factor receptor–tyrosine kinase inhibitors (EGFR-TKIs) like gefitinib, used in cancer treatment [84]. Moreover, *Ocimum basilicum* extract was tested for anticancer activity against the human papillomavirus (HPV)-positive cervical cancer cell lines CaSki and HEK 293, in combination with cisplatin. Interferon-gamma (IFN-γ) secretion was increased, indicating a strong immunomodulatory effect. Additionally, G0/G1 phase cell cycle arrest was also induced [85].

There were not many clinical studies on the anticancer activity of basil extracts. In a recent study from Nomura and colleagues, patients suffering from differentiated thyroid cancer and receiving radioactive iodine therapy after total thyroidectomy were drinking tea prepared from *Ocimum tenuiflorum* leaves after each meal, for 4 days after therapy. Significantly lower rates of both State Anxiety and Trait Anxiety (STAI score), cariogenic bacteria (causing acute sialadenitis after radiotherapy), protein, ammonia, and occult blood were found in the group of tea drinkers, as compared to the control group drinking distilled water. Additionally, the rate of change in the washout ratio for salivary gland scintigraphy was significantly higher in the group drinking basil tea. These findings indicate a protective role of basil tea in the oral mucosa and a reduction in patients’ anxiety after radiotherapy with iodine [86].

In conclusion, basil extracts contain bioactive components with strong anticancer activity, which induce cell cycle arrest, apoptosis, or necrosis, and act synergistically with anticancer drugs. 

## 4. Discussion

**Figure 1:**
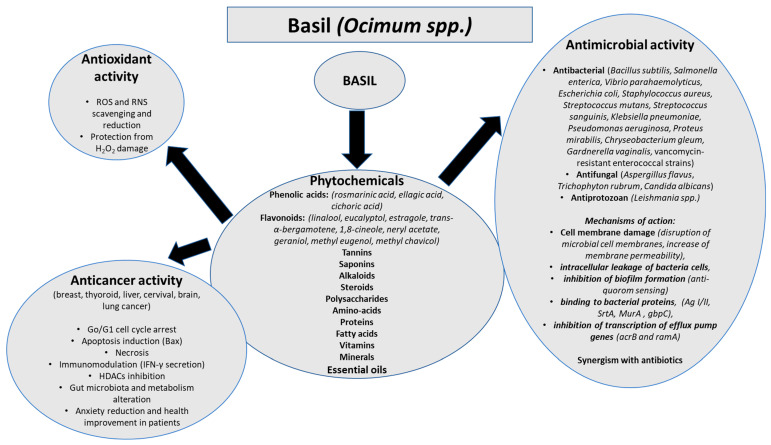
Antioxidant, antimicrobial, and anticancer activities of basil.

Herbs and spices were extensively used many years ago due to their flavor and aroma in cuisine. Additionally, their phytochemical constituents have been reported for their health benefits. Recent research on natural products as agents for prevention, disease therapy, and health promotion has been extensively conducted. Basil is a herb used in many countries, which was reported to have beneficial effects due to its phytochemicals. In this review, we made a comprehensive presentation of the recent literature on the antioxidant, antimicrobial, and anticancer properties of basil, as shown in Figure 1.

Antioxidant activity of basil is dependent on its concentration in phenolic acids (hydroxycinnamic, quininic, betulinic, chicoric, rosmarinic, and ellagic acid), flavonoids (liquiritigenin, catechin, umbelliferone, methylcinnamate, β-linalool, methyl-eugenol, eugenol, estragole, luteolin, luteolin-7-O-glucoside, cymaroside, terpineol, gingerol, linalool, methyl linolenate, α-bergamotene, α-cadinol, tau-cadinol, and kaempferol), terpenes (monoterpenes, sesquiterpenes, and triterpenes) hydrocarbons, phthalates, phytosterols, fatty acids (methyl palmitate, palmitic acid, and linolenic acid), alkaloids, anthocyanins, lignin, coumarin, amino acids, and vitamins (vitamin C). These components have been extracted from leaves or seeds by using solvents (such as ethanol, dichloromethane, acetonitrile, and sunflower oil), purified, and measured by many different methods, such as UPLC-DAD, HPLC-DAD/ESI-ToF-MS, ESI-HRMS/MS, LC-ESI-MS/MS, LC-ESI-QTOF-MS/MS, HS-SPME, and GC-MS. Hydrodistillation and steam distillation were used for extracting essential oils, containing mainly camphor, citral, estragole, and caryophyllene. Total phenolic content was measured by the Folin–Ciocalteu assay. Differences in the quality and content of bioactive components have been noticed between different solvents used. Ethanolic and acetonic extracts have been characterized for higher concentrations of bioactive components, which makes ethanol and acetone useful solvents. All purification methods were characterized for clear results, and are strongly recommended for deep analysis. Additionally, essential oils have also been of great interest, due to the high variety and concentrations of bioactive components included. This makes them suitable candidates for health-promoting effects and the prevention of human diseases. Additionally, different parts of the herb (leaves, seeds, etc.) provide different levels and varieties of bioactive components. Leaf and seed extracts are usually rich in essential oils and bioactive components and can be useful for health promotion. Finally, comparative studies of *Ocimum basilicum* and other species or varieties of basil show differences in concentration and variety of bioactive components. As a conclusion, choosing the suitable part of the plant, the method of extraction and purification, and the solvent, depends on the type of compounds wanted (polar or non-polar, flavonoids, phenolic acids, polysaccharides, amino acids, vitamins, etc.) and the concentration wanted. Bioactive components or essential oils extracted, and their concentration affects the beneficial effects on health, as discussed in this review article.

Bioactive components of basil have been reviewed to serve as protectors from oxidative stress, by reducing ROS and RNS, and offering cytoprotection in cells after H_2_O_2_ treatment or CCI-induced oxidative stress. The antioxidant activity is measured by many different assays, such as the radical-scavenging assays DPPH, ABTS, FRAP, ascorbic acid, and hydrogen peroxide radical scavenging, by measuring the increase in the activity of SOD, CAT, GPX, GR, and levels of GSH, the reduction in MDA, urea, creatinine, TBARS, protein carbonyl content, and lipid peroxidation of cells. All these methods measure different parameters of antioxidant activity. However, different methods show different results, for example, different IC50 levels. It is strongly recommended to use various antioxidant assays in combination and compare results. Additionally, bioactive components content and antioxidant activity have a linear correlation, and depend on the extraction solvent used, with ethanolic, acetonic, dichloromethane, and ethyl acetate extracts showing high levels of phenolic, flavonoid, and tannin content, resulting in high antioxidant activity. More to the point, nanoemulsions of basil essential oil resulted in higher antioxidant activity in a dose-dependent manner, due to their improved water solubility, stability, volatility, and bioavailability. This makes nanoemulsions and microemulsions of basil extracts a strong vehicle for the transfer of the herb’s bioactive components, in future in vivo and clinical trials. Additionally, comparative studies of *Ocimum basilicum* and other *Ocimum* species showed a higher antioxidant activity of *O. basilicum*. As a conclusion, basil extract contains many bioactive components with high antioxidant activity, varying among different extraction and purification methods, different solvents used, and different *Ocimum* species. It is strongly recommended to select basil parts and extraction solvents, as well as a method that obtains high bioactive component concentrations, resulting in high antioxidant activity.

The antimicrobial (antibacterial, antiparasitic, and antifungal) activity of basil (seeds and leaves) against a series of pathogenic and foodborne microorganisms is dependent in bioactive components such as carbohydrates, proteins, saponins, quinones, tannins, alkaloids, phenolic acids, flavonoids (nevadensin), fatty acids (lauric acid), and biocomponents contained in the essential oils, including monoterpene, sesquiterpene, and phenylpropene derivatives, methyl trans-cinnamate, estragole, eugenol, and linalool. Different parts of basil, such as leaves, seeds, etc., have been used in order to extract different bioactive components with high antimicrobial activity. Disk and zone inhibition assays, tube microdilution assay, MIC, MBC, MFC, and LC50 measurements were performed. Bacteria strains tested and inhibited by basil extracts include *Bacillus subtilis, Salmonella enterica*, *Vibrio parahaemolyticus*, *Escherichia coli*, *Staphylococcus aureus*, *Streptococcus mutans*, *Streptococcus sanguinis*, *Klebsiella pneumoniae*, *Pseudomonas aeruginosa*, *Proteus mirabilis*, *Chryseobacterium gleum*, *Gardnerella vaginalis*, *Clostridium perfringens*, and vancomycin-resistant enterococcal strains, whereas growth inhibition of the fungi strains *Aspergillus flavus*, *Trichophyton rubrum*, and *Candida albicans*, as well as the antiprotozoan activity against *Leishmania*, have also been reported. Mechanisms of antimicrobial activity include disruption of microbial cell membranes, increase in membrane permeability, intracellular leakage of bacterial cells, and the inhibition of biofilm formation (anti-quorum-sensing). Molecular mechanisms of these effects include the binding of bioactive components to several bacterial proteins, such as Ag I/II, SrtA, MurA, gbpC, and inhibition of transcription of efflux pump genes (*acrB* and *ramA*). Ethanolic, methanolic, hydroalcoholic, and acetonitrile extracts showed the highest antimicrobial activity in a dose-dependent manner. Usage of these solvents, in order to make antimicrobial agents with basil bioactive components, and their use in the food industry, is strongly recommended. Additionally, essential oils, containing different bioactive components, have also been reported and used for their high antimicrobial effects. In addition to that, microemulsions of essential oils and lignin nanoparticles enhanced this activity, and they are strongly recommended for antimicrobial activity and usage in the food industry. More to the point, essential oils of basil in combination with antibiotics like imipenem enhance the antibacterial activity by synergism, whereas combination with other antibiotics like ciprofloxacin shows an antagonistic effect in some strains, and synergism in others, as shown by in vitro bacteria culture studies and in vivo studies using rat and pig models. These findings make the essential oils of basil suitable candidates as natural preservatives in foods. Finally, basil bioactive components such as dihydrokaempferol-3-glucoside, apigenin-7-glucuronide, and aesculetin have been reported to bind and inhibit the main protease of SARS-CoV-2. However, the antiviral activity of basil needs more research. As a conclusion, basil bioactive components are characterized by a high antimicrobial activity, especially in combination with chemical antimicrobial agents, and can treat infections of MDR bacteria and microorganisms with high infectivity. Usage of these extracts and essential oils in the food industry, or as natural antimicrobial agents in pharmacy, due to their safety aspects, has gained more interest in recent years.

The bioactive components of basil, like flavonoids (estragole), phenolic acids, anthraquinones, tannins, anthocyanins, amino acids, proteins, glycosides, reducing sugars, polysaccharides, and volatile oils, have been reported for their anticancer activity, as shown in many studies for many types of cancer, like breast, thyroid, liver, cervical, brain, and lung. Aqueous, ethanolic, and other organic extracts with anticancer activity have been reported. In vitro studies show inhibition of cell growth and proliferation, induction of Go/G1 cell cycle arrest, apoptosis induction (by inducing apoptotic proteins like Bax), necrosis induction, cytotoxicity, and immunomodulation (as shown by IFN-γ increase in secretion). Τhese activities have been reported for many bioactive components in aqueous and organic extracts of basil. Moreover, secreted extracellular vesicles from basil leaf cells served as a drug delivery system and induced cytotoxicity of cancer cells in vitro. In vivo studies show inhibition of cytokine activity and vitality improvement of cancer patients. Basil tea consumption of *O. tenuiflorum* reduces patients’ anxiety after radiotherapy and protects them from radiotherapy outcomes. Additionally, a synergism of basil polysaccharides with chemotherapeutic drugs like gefitinib in lung tumor growth has been shown in xenograft mouse models. Synergism of basil bioactive components has been reported for cisplatin, too. Mechanisms of anticancer activity include gut microbiota and metabolism modulation, resulting in cancer cell signaling inhibition. Polysaccharides of basil also bind and inhibit HDACs. These findings strongly recommend basil extracts, pure bioactive components, or essential oils as anticancer drugs or food additives, due to their safety and lack of unwanted effects, in comparison to chemotherapeutic drugs. In conclusion, basil bioactive components show high anticancer activity and enhance the cytotoxic and cytostatic activity of chemotherapy.

## 5. Conclusions

Basil, with the most representative species *Ocimum basilicum*, is rich in many bioactive components, including phenolic acids, flavonoids, anthocyanins, tannins, amino acids, proteins, fatty acids, polysaccharides, and vitamins. Basil essential oil is also rich in many of these volatile components. These compounds have been mentioned for their health benefits, including antioxidant, antimicrobial, and anticancer activity. These advantages make basil a rich source of natural products that can be used as food additives or drugs, for preserving or treating many diseases and pathological conditions. However, although there are many in vitro studies mentioning their importance in human health, few clinical studies have been conducted. More in vitro and especially in vivo and clinical studies must be prepared in order to unravel the safety and significance of basil bioactive components on human health.

## Figures and Tables

**Table 1:** Antioxidant, antimicrobial, and anticancer activities of basil.

Research Article (First Author)	Study	Methods	Bioactive Components	Antioxidant Activity	Antimicrobial Activity	Anticancer Activity
Vijay et al., 2025 [40]	In vitro	Hydroalcoholic extraction of Ocimum basilicum leaves. GC-MS Phytochemical tests. DPPH, reducing power, H_2_O_2_ scavenging, and anti-lipid peroxidation assays. Poison food assay, time-killing curve assay.	-	Different IC50 values of antioxidant activity in different methods.	A dynamic interaction between the extract and microbial strain has been reported. Concentration-dependent antifungal activity.	-
Wójciak et al., 2024 [41]	In vitro	Polyphenolic fraction isolated from O.basilicum. UPLC-DAD-MS DPPH, FRAP, SOD, and CAT activity, and MDA levels of human normal colon epithelial and human colorectal adenocarcinoma cells after H_2_O_2_ treatment.	Caffeic acid derivatives (rosmarinic and chicoric acids).	High radical scavenging activity. Prevention of SOD and CAT depletion. MDA levels decrease. Protection of H_2_O_2_-induced cytotoxicity in human normal colon epithelial cells.	-	-
Nadeem et al., 2022 [42]	In vitro	Basil extracts by using n-hexane, dichloromethane, ethanol, and water at three different growth stages (GS), i.e., GS-1 (58 days of growth), GS-2 (69 days of growth), and GS-3 (93 days of growth). LC-ESI-MS/MS DPPH, FRAP, and H_2_O_2_ assays.	Higher concentration of phenolic acid, flavonoids, and tannin content in ethanolic extracts. Rosmarinic acid, ellagic acid, catechin, liquiritigenin, and umbelliferone.	Ethanolic extracts exhibited the highest antioxidant activity.	-	-
Vidaković et al., 2024 [43]	In vitro	Basil aerial parts were extracted using ethanol, dichloromethane, and sunflower oil separately. Re-extraction of the extracted oil with acetonitrile was also prepared. HPLC-DAD/ESI-ToF-MS DPPH assay. Antimicrobial activity was explored in 8 bacterial, 2 yeast, and 1 fungal species by using the broth microdilution method.	In total, 109 compounds were identified in ethanolic, dichloromethane, and acetonitrile extracts. Fatty acids were present in all extracts. Phenolic acids and flavonoids were dominant in ethanolic extracts. Triterpenoids were dominant in dichloromethane and diterpenoids in acetonitrile extracts.	Ethanolic and acetonitrile extracts showed significant radical scavenging potential.	All extracts exhibited high antifungal activity, as compared to the antimicrobial drug nistatin. Antibacterial activities were notable for ethanolic and acetonitrile extracts, whereas the dichloromethane extract showed no activity against bacteria.	-
Abdel-Razakh et al., 2024 [44]	In vitro	Ethanolic, ethyl acetate extracts, and solvent fractions. Total phenolic content (TPC), total flavonoid content (TFC), and total tannin content (TTC). LC-MS. HPLC-ABTS. DPPH and ABTS.	TPC of *O. basilicum* ranged from 64.70 ± 5.2 to 411.16 ± 8.11 mgGAE (gallic acid equivalents) /g DW (dry weight). The ethyl acetate extract obtained the maximum TPC, TFC, and TTC. Rosmarinic acid was identified as the major component in all extracts and all fractions of *O. basilicum*.	Ethyl acetate fractions have the strongest antioxidant activities.	-	-
Mahmoud et al., 2022 [45]	In vitro	Ocimum basilicum residues were dried in an oven and in a microwave. HS-SPME. GC-MS. LC-MS/MS. Folin–Ciocalteu photochemiluminescence.	In total, 30 volatiles were identified in raw material, with β-linalool, methyleugenol, methylcinnamate, and estragole identified as the predominant molecules. A total of 24 and 18 volatiles were detected in the oven- and microwave-dried samples, with a significant decrease in methyleugenol content. The highest TPC was found in the microwaved waste. In total, 8 phenolic acids and 9 flavonoids were identified, with significant contents of rosmarinic acid and luteolin (1042.45 and 11.68 µg/g of dry matter, respectively) in the microwaved samples.	The highest radical scavenging ability was achieved for microwaved waste.	-	-
Qamar et al., 2023 [46]	In vitro and ex vivo	Basil leaf extracts. GC-MS of non-polar extracts. ESI-HRMS/MS of polar extracts. TPC and TFC were measured spectrophotometrically and calculated as GAE/ g DW and rutin equivalent (RE)/g DW, respectively. DPPH, AAPH, and ABTS assays.	In total, 75 compounds (monoterpenes, hydrocarbons, sesquiterpenes, triterpenes, phyto-sterols, and phthalates) were found in non-polar extracts. High fatty acid concentrations. Predominant compounds: linalool (7.65%), terpineol (1.42%), tau-cadinol (13.55%), methyl palmitate (14.24%), palmitic acid (14.31%), linolenic acid (1.30%), and methyl linolenate (17.72%). Alkaloids, phenolic acids, amino acids, coumarins, lignins, flavonoids, and terpenes were identified in polar extracts. The highest TPC and TFC were found in ethyl acetate extract (9.40 mg GAE/g and 15.9 mg RE/g of dry weight, respectively.	All the extracts showed significant antioxidant activity in DPPH and ABTS assays. Dichloromethane extract showed the highest DPPH scavenging activity, i.e., 64.12% ± 0.23 at a concentration of 4 mg/mL. All the extracts inhibited AAPH-induced oxidation in human erythrocytes, being 69.24% ± 0.18 in the dichloromethane extract, 64.44% ± 0.04 in ethyl acetate, and 53.33% ± 0.09 in the acetone extract.	-	-
Siripongvutikorn et al., 2024 [47]	In vitro	Spicy basil leaf extracts LC-ESI-QTOF-MS/MS DPPH, FRAP, ABTS, and ORAC.	62 components, including quininic acid, hydroxycinnamic acid, luteolin, kaempferol, catechin, eugenol, betulinic acid, and gingerol.	High antioxidant activity, mainly depending on cynaroside and luteolin-7-O-glucoside.	-	-
Złotek et al., 2016 [48]	In vitro	Fresh, frozen, and lyophilized basil leaves. Extraction with acetone or methanol, with/without the addition of acetic acid in different steps. Folin–Ciocalteau, DPPH, Reducing power assays.	Highest TPC in acetone mixtures with the highest addition of acetic acid of fresh and freeze-dried material. The three-fold procedure was more effective than the once-shaking procedure in most of the extracts obtained from fresh basil leaves, unlike the extracts from frozen material. No differences in the TPC between the two procedures used in the lyophilized basil leaves.	Positive correlation between TPC and antioxidant activity.	-	-
Yibeltal et al., 2022 [50]	In vitro	*Ocimum basilicum* leaf and flower essential oils extraction. DPPH and H_2_O_2_ free radical scavenging assays. Disk diffusion, broth dilution assays.		Higher DPPH scavenging activity (86.45%) for leaf oil extract.	The strongest antibacterial activity with maximum zone of inhibition (15.47 mm), MIC (0.09 μg/mL, and MBC (0.19 μg/mL) was exhibited by the flower oil extract against *Staphylococcus aureus* ATCC-25923. The strongest antifungal activity with maximum zone of inhibition (15.90 mm), MIC (0.125 μg/mL), and MFC (0.09 μg/mL) was recorded for leaf oil against *Candida albicans*.	-
Eid et al., 2023 [51]	In vitro	*Ocimum basilicum* seed from Palestine. Essential oil extraction. DPPH. Broth microdilution assay, cytotoxicity assay.		IC50 of 23.44 ± 0.9 µg/mL compared with trolox (IC50 2.7 ± 0.5 µg/mL).	Antibacterial activity against *Klebsiella pneumoniae*, *Escherichia coli*, *Staphylococcus aureus*, *Proteus mirabilis*, and *Pseudomonas aeruginosa*, and antifungal activity against *Candida albicans*.	*O. basilicum* seed EO showed high anticancer activity against Hep3B (IC50 56.23 ± 1.32 µg/mL) and MCF-7 (80.35 ± 1.17 µg/mL), as compared to doxorubicin.
Park et al., 2025 [52]	In vitro	Basil essential oil extraction. HPLC, GC-MS, DPPH, and ABTS assays. Disk diffusion test, broth microdilution assay (MIC).	Monoterpene, phenylpropene, and sesquiterpene derivatives. Methyl trans-cinnamate was identified as the major compound in fraction 3 obtained by preparative HPLC.	DPPH and ABTS assays returned EC50 values of 115.36 (DPPH) and 54.77 (ABTS) µg/mL.	Significant antimicrobial activity against *Gardnerella vaginalis*, *Fannyhessea vaginae*, *Chryseobacterium gleum*, and *Candida albicans*, with inhibition zones of up to 25.88 mm and MIC values ranging from 31 to 500 µg/mL. Fraction 3 showed the highest antimicrobial activity.	-
Li et al., 2017 [53]	In vitro	Sweet basil crude oil was processed via molecular distillation. GC-MS. DPPH and ABTS assays.	Major constituents of the residue fraction: estragole (17.06%), methyl eugenol (11.35%), and linoleic acid (11.40%). Major constituents of the distillate fraction: methyl eugenol (16.96%), α-cadinol (16.24%), α-bergamotene (11.92%).	The residue fraction markedly scavenged the DPPH (IC50 = 1.092 ± 0.066 mg/mL) and ABTS (IC50 = 0.707 ± 0.042 mg/mL) radicals.	-	-
Sharma et al., 2022 [54]	In vitro	Leaf extracts from dried powder of *Ocimum basilicum* (Green tulsi), *O. gratissimum* (Jungli tulsi), and *O. tenuiflorum* (Black tulsi) were prepared by using acetone, ethanol, methanol, and water. Folin–Ciocalteu (TPC), AlCl_3_ assay (TFC), and total condensed tannin analysis. Fingerprint analysis using UV, Fourier transform infrared (FT-IR), and fluorescent spectroscopy, DPPH, and total antioxidant capacity (TAC) assay.	High levels of polyphenolics were detected in all the solvent extracts. Acetone provided the highest concentrations of phenolics, flavonoids, and tannins in all *Ocimum* species. *O. tenuiflorum* showed the maximum level of antioxidants.	*O. tenuiflorum* showed the highest antioxidant activity.	-	-
Araújo Couto et al., 2019 [55]	In vitro	Essential oils extraction of 24 different basil genotypes. DPPH and linoleic acid peroxidation assays.	Eugenol was the major component identified.	A total of 9 essential oils exhibited high antioxidant potential, with at least 52.68% of inhibition of the linoleic acid peroxidation at 10 µL/mL and 76.34% of inhibition of the DPPH radical at 1 µL/mL. The major compound eugenol had the highest antioxidant activity. Synergism between minor compounds results in high antioxidant activity.	-	-
Fayezizadeh et al., 2023 [56]	In vitro	In total, 21 cultivars and genotypes of basil microgreen extracts. Folin–Ciocalteu (TPC), AlCl_3_ assay (TFC), anthocyanin, and vitamin C content measurement. DPPH and APCI.	Variation in phytochemical components between basil genotypes.	The highest APCI was measured in the Persian Ablagh genotype (70.30). In total, 21 basil genotypes were classified into 4 clusters: cluster 1 (lowest antioxidant capacity and TPC), cluster 2 (lowest anthocyanin, vitamin C, and APCI), cluster 3 (highest vitamin C, TPC, antioxidant capacity, and APCI), and cluster 4 (highest levels of anthocyanin). The average annual temperature of the origin of basil seeds plays an important role in the synthesis of antioxidant components. Most of the seeds with moderate origin had a higher APCI.	-	-
Mahendran & Vimolmangkang, 2023 [57]	In vitro	*O. americanum* and *O. basilicum* EOs extraction by steam distillation in a Clevenger-type apparatus. GC-MS DPPH, FRAP, ABTS, and metal-chelating assays. Disk-diffusion test and broth microdilution method.	Camphor (33.869%), limonene (7.215%), longifolene (6.727%), caryophyllene (5.500%), and isoledene (5.472%) were the major compounds in *O. americanum* leaf EO. The EO yield was 0.4%, and citral (19.557%), estragole (18.582%), camphor (9.224%), and caryophyllene (3.009%) were the major compounds found among the 37 chemical constituents identified in *O. basilicum* EO.	*O. basilicum* exhibited a more potent antioxidant activity than *O. americanum.*	Zone of inhibition and MIC of the Eos in the disk diffusion and the microdilution methods were 8.00 ± 0.19 mm to 26.43 ± 2.19 mm and 3.12–100 µg/mL, respectively.	-
Neeharika et al., 2025 [58]	In vitro	Ethanolic, methanolic, and distilled water extracts of *O. gratissimum* and *O. basilicum* seeds. ABTS, O_2_^−^, lipid peroxidation assays. Agar well diffusion, fungal biomass inhibition assays	Ethanolic extracts of germinated *O. gratissimum* and *O. basilicum* seeds exhibited higher concentrations of phenols (21.03 ± 0.01 mg GAE/g and 21.46 ± 0.01 mg GAE/g, respectively) and flavonoids (11.92 ± 0.03 mg quercetin equivalent (QE)/g and 14.45 ± 0.04 mg QE/g, respectively), as compared to other extracts.	Ethanolic extracts of germinated *O. gratissimum* and *O. basilicum* exhibited IC50 values for scavenging ABTS (0.013 ± 0.00 mg/mL and 0.007 ± 0.00 mg/mL, respectively) and superoxide anion radical (4.33 ± 0.01 mg/mL and 4.14 ± 0.00 mg/mL respectively) and for inhibiting lipid oxidation (2.57 ± 0.00 mg/mL and 2.33 ± 0.00 mg/mL, respectively, as compared to other extracts.	Ethanolic extracts of germinated *O. gratissimum* and *O. basilicum* exhibited higher inhibition zones for *Bacillus subtilis* (13.98 ± 0.18 mm, 17.02 ± 0.18 mm, respectively), *Vibrio parahaemolyticus* (19.00 ± 0.20 mm, 22.58 ± 0.45 mm, respectively), *Salmonella enterica* (24.98 ± 0.18 mm, 22.17 ± 0.15 mm, respectively), and *Escherichia coli* (23.50 ± 0.50 mm, 27.00 ± 0.20 mm, respectively) and better inhibition of *Aspergillus flavus* growth (93.28% and 81.77%, respectively), as compared to other extracts.	-
Tenore et al., 2017 [59]	In vitro	Napoletano green and purple basil (*Ocimum basilicum*) varieties extracts. DPPH and FRAP assays. MIC.	Higher polyphenolic concentration as compared to other, more conventional, and geographically different basil varieties.	Napoletano purple basil revealed higher radical-scavenging and ferric-reducing capacities than the green one, probably due to its anthocyanin content.	Both basil varieties exhibited activity against a broad spectrum of food-borne and human pathogenic microorganisms (Gram^+^ and Gram^−^ bacteria, yeasts).	-
Shiwakoti et al., 2017 [60]	In vitro	EO extraction of sweet basil (*Ocimum basilicum*) and holy basil (*Ocimum tenuiflorum*) by steam distillation and hydrodistillation. ORAC	In both basil species, the EO yield was higher from steam distillation than from hydrodistillation. Same compounds were identified in both types of extraction, with differences in concentration. In the EO of *O. basilicum*, the concentration of 74% of the identified compounds was higher in steam distillation as compared to the hydrodistillation, whereas in the EO of *O. tenuiflorum*, the concentration of 84% of the identified compounds was higher in steam distillation. However, the concentrations of estragole and methyl cinnamate in *O. basilicum* EO and methyl eugenol in *O. tenuiflorum* EO were significantly higher in hydrodistillation extracts.	The type of distillation did not affect the antioxidant capacity of basil EO in both species.	-	-
Manzoor et al., 2023 [61]	In vitro	Nanosized lavender, basil, and clove EO in microemulsions. DPPH. Agar well diffusion, broth microdilution assays. The crystal violet assay was used to measure the growth and development of biofilms.	EOs concentration had a great impact on the physicochemical and biological properties of microemulsions.	Dose-dependent antioxidant capacity.	Microemulsions demonstrated more efficient antibacterial activity against *Staphylococcus aureus* and *E. coli*, at concentrations lower than pure EOs.	-
Abidoye et al., 2022 [62]	In vitro	A drink made with roselle calyces (*Hibiscus sabdariffa*) and sweet basil leaves (*Ocimum basilicum*). The roselle–basil samples at different blend ratios were analyzed for pH, total soluble solids, total titratable acidity, vitamin C, lycopene, TPC, antioxidant properties (ABTS, ORAC), and storage stability at different temperatures (4 and 29 °C).		The incorporation of sweet basil leaves to roselle calyces slightly decreased the vitamin C and lycopene content of the drink, whereas they increased the total carotenoid and antioxidant activities. Storing samples at 29 °C resulted in higher antioxidant activity, as compared to storage at 4 °C.		
Othman et al., 2021 [63]	In vivo	Hail *Ocimum* extract and its total flavonoids against hepatorenal damage in experimental diabetes induced by HFD and injection of streptozotocin in rats. Diabetic animals were co-treated daily with HOE, flavonoids, or metformin as a standard anti-diabetic drug for 4 weeks. GSH levels. SOD, CAT, GPX, GR activity.	-	Co-treatment of diabetic animals with Hail *Ocimum* extract or its total flavonoids protected them from oxidative stress induced by HFD and streptozotocin treatment (GSH levels increased, and SOD, CAT, GPX, and GR enzyme activity were induced).	-	-
Eftekhar et al., 2019 [64]	In vivo	Effect of *O. basilicum* on tracheal responsiveness to methacholine and ovalbumin, and bronchoalveolar lavage fluid levels of oxidant-antioxidant biomarkers in sensitized rats. In total, 6 groups of rats, including the control (group C), sensitized rats to ovalbumin (group S), S groups treated with 3 concentrations of *O. basilicum* (0.75, 1.5, and 3 mg/mL), and 1.25 μg/mL of dexamethasone were studied. MDA, TBARS, total stable oxidation products of NO metabolism (NO_2_^−^/NO_3_^−^), and total thiol content. SOD and CAT activity.	-	Decrease in the tracheal bronchoalveolar lavage fluid levels of oxidant markers of rats sensitized to methacholine and ovalbumin, and an increase in antioxidant marker levels, in a dose-dependent manner.	-	-
Ben Mansour et al., 2024 [65]	In vivo	*Ocimum basilicum* seeds methanolic extraction. A single dose of CCl4 was used to induce oxidative stress in rats, which was demonstrated by a significant rise in serum enzyme markers. Extract was administered for 15 consecutive days (200 mg/kg body weight) to Wistar rats before CCl4 treatment. Kidney SOD, CAT, GPX activities and GSH, TBARS, urea, creatinine, lipid peroxidation and protein carbonyl levels were measured.	-	Basil seed extract administration resulted in a significant reduction in urea, creatinine, TBARS, protein carbonyls, and lipid peroxidation levels, whereas antioxidant enzyme activity, like GPΧ, SOD, catalase, and GSH levels, was elevated.	-	-
Khot et al., 2023 [67]	In vitro	Ethanol-based extraction of Ocimum basilicum seed was performed by the Soxhlet method, and aqueous-based extracts by the hot infusion procedure. Extracts were assessed for MIC, MBC, zone of inhibition, and time-kill assay on periodontal pathogens, and compared the effectiveness against 0.12% chlorhexidine gluconate.	-	-	10 mg/mL of ethanolic extract was effective against periodontal pathogens, whereas 4.7 mg/mL of aqueous extract was proven effective. Aqueous extract developed a wider zone against periodontal pathogens compared to ethanolic extract. A statistically significant difference was found in the effectiveness between the extract and chlorhexidine.	-
Alsalamah et al., 2022 [68]	In vitro	Ethanolic and methanolic extracts of *Ocimum basilicum* seeds and leaves. Disk diffusion and direct contact test were used for antibacterial activity measurement against 3 strains. Spectrophotometry.	Methanolic extracts of leaves contained tannins, flavonoids, glycosides, and steroids, whereas seed extracts contained saponins, flavonoids, and steroids. Stems contained saponins and flavonoids.	-	The plant extracts inhibited *Staphylococcus aureus*, *Pseudomonas aeruginosa*, and *E. coli*. Leaf extracts were more potent than seed and stem extracts.	-
Backiam et al., 2023 [69]	In vitro	*Ocimum basilicum* leaf extract was prepared using ethanol, methanol, and water. GC-MS. Folin–Ciocalteu (TPC) and AlCl_3_ (TFC). Antibacterial activity against vancomycin-resistant (VRE) enterococcal strains and microbial type culture collection (MTCC) strains. Agar well-diffusion assay and MIC. DNA and protein absorption at 260 nm, scanning electron microscopy (SEM).	The ethanol and methanol extracts contain alkaloids, flavonoids, phenolic compounds, tannins, saponins, quinones, carbohydrates, and proteins, except for steroids and terpenoids. In addition to steroids and terpenoids, tannin was also absent in the aqueous extract. Total phenolic and flavonoid content was high in ethanolic, followed by methanolic and aqueous extracts. Ethanolic extracts included 19 compounds.	-	Ethanolic and methanolic extracts showed strong antimicrobial activity against VRE and MTCC strains at a concentration of 20 mg/mL, as compared to the aqueous extract. *Staphylococcus aureus* is highly susceptible to ethanolic extract at a concentration of 8 mg/mL and followed by other MTCC strains. Vancomycin-resistant enterococci pathogens were inhibited at the MIC of 14, 16, and 20 mg/mL of ethanolic, methanolic, and aqueous extracts, respectively. The loss of cell membrane integrity and cell membrane damage were the effective mechanisms of plant extract antimicrobial activity.	-
Yaldiz et al., 2023 [70]	In vitro	Essential oil and ethanol extract of basil. Agar well, disk, and agar well diffusion assays. Anti-quorum-sensing activity and violacein pigment isolation by spectrophotometric analysis. Biofilm biomass measurement.	-	-	EO and ethanol extracts of basil exhibited both antibacterial activity and anti-quorum-sensing activity against Gram-negative and Gram-positive bacterial species, and antifungal effects on *C. albicans*. Among the tested microorganisms, the genotypes of PI 531396, PI 296390, PI 414199, PI 253157, PI 296391, PI 652071, midnight, and Dino cultivars have been found to be more susceptible than other genotypes. The highest effect on the quorum-sensing system was found in Moonlight and Dino cultivars, PI 296391, PI 414199, PI 652070, PI 172997, and PI 190100 genotypes. Dendrogram analysis has shown that there is a relationship between different genotypes, depending on microorganisms and anti-quorum-sensing activity. Ames 29184, PI 207498, and PI 379412 genotypes were clustered in the same group.	-
Putri et al., 2023 [71]	In vitro and in silico	Nevadensin (a flavonoid of basil) is an antibacterial against *S. mutans*. Disk diffusion and micro-dilution assays. Ligand–protein docking.	-	-	MIC and MBC values of nevadensin are 900 and 7200 μg/mL, respectively. The binding energy of nevadensin to SrtA, gbpC, and Ag I/II genes was −4.53, 8.37, and −6.12 kcal/mol, respectively.	-
Herdiyati et al., 2021 [72]	In vitro and in silico	Fresh leaves from *O. americanum* were extracted with n-hexane and purified by a combination of column chromatography on normal and reverse phases, together with in vitro bioactivity assay against *S. mutans* ATCC 25175 and *S. sanguinis* ATCC 10556, respectively, while in silico molecular docking simulation of lauric acid was conducted using PyRx 0.8.	-	-	The structure determination of an antibacterial compound by spectroscopic methods resulted in an active compound, lauric acid. The in vitro evaluation of antibacterial activity in lauric acid showed MIC and MBC values of 78.13 and 156.3 ppm and 1250 and 2500 ppm against S. sanguinis and S. mutans, respectively. In silico evaluation determined lauric acid as a MurA enzyme inhibitor. Lauric acid showed a binding affinity of −5.2 Kcal/mol, which was higher than fosfomycin.	-
Araújo Silva et al., 2016 [73]	In vitro	*O. basilicum* leaves extraction by steam distillation. Broth microdilution (MIC). Combinations of *O. basilicum* oil with ciprofloxacin or imipenem were analyzed by the checkerboard method, where fractional inhibitory concentration (FIC) indices were calculated.	-	-	*O.basilicum* EO, imipenem, and ciprofloxacin showed respective MIC antibacterial activities of 1024, 4, and 2 μg/mL against *S. aureus*. In *S. aureus*, the oil with imipenem association showed a synergistic effect (FIC = 0.0625), while the oil with ciprofloxacin showed antagonism (FIC value = 4.25). In *P. aeruginosa*, the imipenem/oil association showed an additive effect for ATCC strains, and synergism for the clinical strain (FIC values = 0.75 and 0.0625). The association of *O. basilicum* EO with ciprofloxacin showed synergism for clinical strains (FIC value = 0.09).	-
El-Samahy et al., 2024 [74]	In vitro and in vivo	*O. basilicum* lignin nanoparticles were tested for their antimicrobial potential against *Escherichia coli*, *Enterococcus faecalis*, *Klebsiella pneumoniae*, *Staphylococcus aureus*, *Salmonella enterica*, *Trichophyton mentagrophytes*, *Trichophyton rubrum*, and *Microsporum canis*, and further tested for their anti-efflux activity against ciprofloxacin-resistant *Salmonella enterica* strains and for treating *Salmonella* infection in a rat model. Antifungal efficacy for treating *T. rubrum* infection in a guinea pig model was also tested. Disk diffusion and broth microdilution assays. Real-time PCR (polymerase chain reaction).	-	-	Nanoparticles showed antibacterial activity against *Salmonella enterica* species with a MIC range of 0.5–2 µg/mL and antifungal activity against *T. rubrum* with a MIC range of 0.25–2 µg/mL. Downregulation of the expression of ramA and acrB efflux pump genes (fold change values ranged from 0.2989 to 0.5434—0.4601 to 0.4730 for ramA and 0.3842–0.6199; 0.5035–0.8351 for acrB) was also obtained. Oral administration of basil lignin nanoparticles in combination with ciprofloxacin had a significant effect on all blood parameters, as well as on liver and kidney function parameters. Oxidative stress mediators, total antioxidant capacity, and MDA were abolished by oral administration of basil lignin nanoparticles (0.5 mL/rat once daily for 5 days). INF-γ and TNF-α were also reduced in comparison with the positive control group and the ciprofloxacin-treated group. Histopathological examination of the liver and intestine of basil lignin nanoparticles-treated rats revealed an elevation in *Salmonella* clearance. Treatment of *T. rubrum*-infected guinea pigs with basil lignin nanoparticles topically in combination with itraconazole resulted in a reduction in lesion scores, microscopy, and culture results.	-
Khan et al., 2015 [75]	In vitro	Antileishmanial, antibacterial, and brine lethality assays of the leaf extract of *O. basilicum* from the Peshawar region. In total, 6 Gram^+^ and 6 Gram^−^ were tested. Negative bacteria. Brine shrimp cytotoxicity assay.	-	-	LC50 = 21.67 µg/mL for *Leishmania.* Significant inhibitory activity at the highest two concentrations, ranging from 20.66 ± 0.31 to 31.86 ± 0.80 for Gram^+^ strains *Clostridium perfringens* type C and *Bacillus subtitilis*, respectively, as compared to gentamycin (27.36 ± 0.55 and 21.80 ± 0.72, respectively). Good activity was observed Gram^-^ strains. The highest zone of inhibition was recorded for *Pseudomonas aeroginosa* (28.83 ± 0.28) at the highest concentration (10 mg/mL). The LC50 value obtained for the brine shrimp lethality assay was 91.56 µg/mL.	-
Kurnia et al., 2023 [76]	In silico	Inhibition of the main protease of SARS-CoV-2 by apigenin-7-glucuronide, dihydrokaempferol-3-glucoside, and aesculetin from *O. basilicum.* Autodock 4.0 tools were used for the prediction of the molecular docking inhibition mechanism, such as the pkcsm and protox online web server for ADMET analysis and drug likeness.	-	-	The binding affinity for apigenin-7-glucuronide was −8.77 Kcal/mol, for dihydrokaempferol-3-glucoside −8.96 Kcal/mol, and for esculetin −5.79 Kcal/mol. Inhibition constant values were 375.81 nM, 270.09 nM, and 57.11 µM, respectively. Apigenin-7-glucuronide and dihydrokaempferol-3-glucoside bind to the main protease enzymes on the active sites of Cys145 and His41, while aesculetin only binds to the active sites of Cys145. On ADMET analysis, all 3 compounds met the predicted pharmacokinetic parameters, although there are some specific parameters that must be considered, especially for aesculetin compounds. For drug-likeness analysis, apigenin-7-glucuronide and dihydrokaempferol-3-glucoside compounds have one violation, and aesculetin has no violations.	-
Avcibasi et al., 2023 [78]	In vitro	Estragole isolation from basil leaves via ethanolic extraction using an 80% ethanol concentration and HPLC. Estragole was radiolabeled with ^131^I using the iodogen method. Quality control studies were carried out by using TLRC. Next, in vitro cell culture studies were performed to investigate the bio-affinity of with human medulloblastoma (DAOY) and human glioblastoma-astrocytoma (U-87 MG) cell lines. Finally, the cytotoxicity was determined, and cell uptake was investigated on cancer cell lines by incorporation studies.	-	-	-	^131^I-estragole has a significant uptake in the brain cancer cells.
Alkhateeb et al., 2021 [79]	In vitro	Fresh dark purple blossoms of basil were extracted at low temperature (0 °C) using a watery solvent. Human MCF7 breast cancer cells were then treated with 3 separate fluctuated concentrations of 0, 50, 150, and 250 µg/mL for 24 and 48 h. Mitochondrial fission contributed to the induced apoptosis, which was evaluated.	Anthocyanins, anthraquinones, tannins, reducing sugars, glycosides, proteins, amino acids, flavonoids, and volatile oils were detected, whereas terpenoids and alkaloids were absent. A frail presence of steroids in basil blossom aqueous concentrate was noticed.	-	-	Glucose uptake was alleviated in a dose-dependent manner in MCF7 cells with the extract induced for 24 h, resulting in mitochondrial fission and apoptosis induction.
Chintapula et al., 2024 [80]	In vitro	Extracellular vehicles purified from apoplast washing fluid of basil leaves were used as a therapeutic agent against cancer. Characterization of extracellular vehicles revealed a size range of 100–250 nm, which were later assessed for their cell uptake and apoptosis-inducing abilities in pancreatic cancer cell line MIA PaCa-2. Cell viability and clonogenic assays. Reverse-Transcription (RT)-PCR and Western blotting.	-	-	-	Basil extracellular vehicles showed a significant cytotoxic effect on pancreatic cancer cell line MIA PaCa-2 at a concentration of 80 and 160 μg/mL in cell viability, as well as clonogenic assays. Similarly, RT-PCR and Western blot analysis have shown upregulation in apoptotic gene and protein expression of Bax, respectively, in BasEV treatment groups compared to untreated controls of MIA PaCa-2.
Torres et al., 2018 [81]	In vitro	The unfractionated aqueous leaf extracts of *O. basilicum* and *O. gratissimum* were chemically characterized and tested for their cytotoxic, cytostatic, and anti-proliferative properties against the human breast cancer cell line MCF-7.				Both extracts presented cytostatic effects with an 80% decrease in MCF-7 cell growth at 1 mg/mL. However, only *O. basilicum* extract promoted cytotoxicity, interfering with the cell viability even after interruption of the treatment, and affected the cell proliferation and metabolism, as evaluated in terms of lactate production and intracellular ATP content. After 24 h of treatment, *O. basilicum* extract-treated cells were induced for apoptosis, whereas *O. gratissimum* extract-treated cells were induced for necrosis. The treatment with both extracts activated AMPK, but *O. basilicum* extract was much more efficient. Only *O. basilicum* extract activated mTOR signaling.
Sharma et al., 2016 [82]	In silico and in vitro	An in silico structure-activity relationship study on orientin from *Ocimum sanctum* L. (*O. tenuiflorum*) was performed, and a pharmacophore mapping and QSAR model was built to screen out the potential structurally similar analogs from the chemical database of Discovery Studio (DSv3.5, Accelrys, USA) as potential anticancer agents. Analog fenofibryl glucuronide was selected for in vitro cytotoxic/anticancer activity evaluation through MTT assay. The binding affinity and mode of action of orientin and its analog were explored through molecular docking studies on quinone oxidoreductase. Cytotoxicity of HepG2 liver cancer cell line.	-	-	-	Only 41% of cell death at 202.389 μM after 96 h of treatment was observed. The selected orientin analog fenofibryl glucuronide was non-cytotoxic/non-anti-carcinogenic up to 100 μg/mL (202.389 μM) concentrations for a long-term exposure in HepG2 cells. Orientin and its analog showed no activity or less cytotoxicity on HepG2 cells.
Bhura et al., 2022 [83]	In silico	Basil polysaccharides were screened against HDAC1-2, 4–8, and HAT (targets for breast cancer) using molecular docking studies along with swissADME studies to check the drug likeliness.	-	-	-	Glucosamine ring, glucosamine linear, glucuronic acid linear, rhamnose linear, glucuronic acid ring, galactose ring, mannose, glucose, and xylose exhibited consistent binding potential against the epigenetic targets (HDAC1, HDAC2, HDAC4, HDAC5, HDAC6, HDAC7, HDAC8, and HAT).
Feng et al., 2024 [84]	In vivo and in vitro	Basil polysaccharides were administered in a gefitinib-resistant xenograft mouse model in order to investigate whether BPS enhances the antitumor effects of gefitinib. A multi-omics approach, including 16S rDNA amplicon sequencing and LC-MS, was used to elucidate these synergistic effects.	-	-	-	Basil polysaccharides can enhance tumor responsiveness to gefitinib by modulating the gut microbiota and its metabolites through multiple metabolic pathways. These changes could potentially affect cancer-related signaling pathways and lung resistance-related proteins, which are pivotal in determining the efficacy of EGFR-TKIs, such as gefitinib, in cancer treatment.
Vaghasia et al., 2025 [85]	In vitro	Extracts from *Ocimum basilicum* were tested on CaSki and HEK 293 HPV-positive cervical cancer cells alongside cisplatin. Cytotoxicity, genotoxicity, cell migration, HPV DNA inhibition, IFN-γ secretion, and cell cycle modulation were assessed using quantitative PCR (qPCR), Enzyme-Linked Immunosorbent Assay (ELISA), and flow cytometry.	-	-	-	*O. basilicum* extract in combination with cisplatin significantly enhanced IFN-γ secretion and induced G0/G1 phase cell cycle arrest.
Nomura et al., 2023 [86]	Clinical study	A total of 44 differentiated thyroid cancer patients after total thyroidectomy were randomly divided into Group A (Basil tea group, n = 22) and Group B (Control group, n = 22). Subjects in Group A drank 180 mL of Basil tea prepared from 2.0 g of holy basil (Ocimum tenuiflorum Linn.) leaves after each meal for 4 days, starting on the day radioactive iodine therapy was performed. Those in Group B drank the same amount of distilled water after each meal for the same period as those in Group A. The State-Trait Anxiety Inventory (STAI) was used to assess anxiety, while the saliva component test and salivary gland scintigraphy were used to assess the oral cavity.	-	-	-	The rate of change in the STAI score was significantly lower in Group A than in Group B (*p* < 0.05). The rates of change in cariogenic bacteria, ammonia, protein, and occult blood were significantly lower in Group A than in Group B (*p* < 0.05). The rate of change of the washout ratio for salivary gland scintigraphy was significantly lower in Group B than in Group A (*p* < 0.05).

## Data Availability

No new data were created or analyzed in this study. Data sharing is not applicable to this article.

## Abbreviations

AAPH	azobis(2-amidinopropane) dihydrochloride
ABTS	2,2′-azino-bis-(3-ethylbenzothiazoline-6-sulfonic acid
ADME	absorption, distribution, metabolism, and excretion
ADMET	absorption, distribution, metabolism, excretion, and toxicity
AMP	adenosine monophosphate
AMPK	AMP-activated protein kinase
APCI	antioxidant potential composite index
CAT	catalase
CCI_4_	carbon tetrachloride
DAD	diode-array detector
DPPH	2,2-diphenyl-1-picrylhydrazyl
DTC	differentiated thyroid cancer
DW	dry weight
EC50	half maximal effective concentration
EGFR-TKIs	epidermal growth factor receptor–tyrosine kinase inhibitors
ELISA	enzyme-linked immunosorbent assay
Eos	essential oils
ESI-HR-MS/MS	electrospray ionization–high-resolution tandem mass spectrometry/mass spectrometry
FIC	fractional inhibitory concentration
FRAP	ferric reducing antioxidant power (FRAP)
GAE	gallic acid equivalent
GC-MS	gas chromatography–mass spectrometry
GPX	glutathione peroxidase
GR	glutathione reductase
Grx	glutaredoxin
GS	growth stage
GSH	reduced glutathione
HAT	histone acetyltransferases
HDAC	histone deacetylases
HDF	high-fat diet
HO^•^	hydroxyl radical
HPLC-DAD/ESI-ToF-MS	high-performance liquid chromatography diode-array detector/electrospray ionization time-of-flight
HPV	human papillomavirus
HS-SPME	headspace solid-phase microextraction
H_2_O_2_	hydrogen peroxide
IC50	inhibitory concentration 50
Ig	immunoglobulin
LC-ESI-MS/MS	liquid chromatography–electrospray ionization-tandem mass spectrometry
LC-ESI-QTOF-MS/MS	liquid chromatography–electrospray ionization-quadrupole time-of-flight-mass spectrometry
LC50	lethal concentration 50
LPS	lipopolysaccharide
MAPK	mitogen-activated protein kinase
MBC	minimum bactericidal concentration
MDA	malondialdehyde
MDR	multidrug-resistance
MFC	minimum fungicidal concentration
MIC	minimum inhibitory concentration
MS	mass spectrometry
MTCC	microbial type culture collection
mTOR	mammalian target of rapamycin
MTT	3-(4,5-di methyl thiazol-2-yl)-2,5-diphenyltetrazolium bromide
NF-kB	nuclear factor kappa-b
O_2_.^−^	superoxide anion
ORAC	oxygen radical absorbance capacity
PCR	polymerase chain reaction
PLA2	phospholipase A2
QE	quercetin equivalent
qPCR	quantitative polymerase chain reaction
QSAR	quantitative structure-activity relationship
RAIT	radioactive iodine therapy
RE	rutin equivalent
RNS	reactive nitrogen species
ROS	reactive oxygen species
RT-PCR	reverse-transcription polymerase chain reaction
SEM	scanning electron microscopy
SOD	superoxide dismutase
STAI	state anxiety and trait anxiety
TAC	total antioxidant capacity
TBARS	thiobarbituric acid reactive substances
TFC	total flavonoid content
TLRC	thin-layer radio chromatography
TPC	total phenolic content
Trx	thioredoxin
TTT	total tannin content
UPLC	ultra-performance liquid chromatography
VRE	vancomycin-resistant
